# Analyses of the Temporal Dynamics of Fungal Communities Colonizing the Healthy Wood Tissues of Esca Leaf-Symptomatic and Asymptomatic Vines

**DOI:** 10.1371/journal.pone.0095928

**Published:** 2014-05-01

**Authors:** Emilie Bruez, Jessica Vallance, Jonathan Gerbore, Pascal Lecomte, Jean-Pierre Da Costa, Lucia Guerin-Dubrana, Patrice Rey

**Affiliations:** 1 Université de Bordeaux, ISVV, UMR1065 Santé et Agroécologie du Vignoble (SAVE), Bordeaux Sciences Agro, Villenave d’Ornon, France; 2 INRA, ISVV, UMR1065 SAVE, Villenave d’Ornon, France; 3 BIOVITIS, Saint Etienne de Chomeil, France; 4 IMS, Université de Bordeaux, Bordeaux Sciences Agro, Talence, France; Virginia Tech, United States of America

## Abstract

Esca, a Grapevine Trunk Disease (GTD), is of major concern for viticulture worldwide. Our study compares the fungal communities that inhabit the wood tissues of vines that expressed or not foliar esca-symptoms. The trunk and rootstock tissues were apparently healthy, whether the 10 year-old plants were symptomatic or not. The only difference was in the cordon, which contained white rot, a typical form of esca, in 79% of symptomatic plants. Observations over a period of one year using a fingerprint method, Single Strand Conformation Polymorphism (SSCP), and the ITS-DNA sequencing of cultivable fungi, showed that shifts occurred in the fungal communities colonizing the healthy wood tissues. However, whatever the sampling time, spring, summer, autumn or winter, the fungi colonizing the healthy tissues of asymptomatic or symptomatic plants were not significantly different. Forty-eight genera were isolated, with species of Hypocreaceae and Botryosphaeriaceae being the most abundant species. Diverse fungal assemblages, made up of potentially plant-pathogenic and -protective fungi, colonized these non-necrotic tissues. Some fungi, possibly involved in GTD, inhabited the non-necrotic wood of young plants, but no increase in necrosis areas was observed over the one-year period.

## Introduction

Grapevine trunk diseases (GTDs) that affect vineyards are of major concern for the wine industry. The three main diseases, eutypa dieback, esca disease and Botryosphaeria dieback, constitute a threat for the main vine-growing regions of the southern and northern hemispheres [1]–[4]. Over the last 15 years, an increase in the incidence of these diseases has been reported worldwide [5]–[10]. In France, until the late nineties, besides eutypa dieback, the most common trunk disease was esca but, at the turn of the century, a third, new disease, a Botryosphaeria dieback also called Black Dead Arm in France, was identified in the French vine-growing areas [11].

In the last decade, the National Grapevine Trunk Diseases Survey was established in France in order to monitor and analyze the importance and severity of the trunk diseases (period 2003 to 2008). It was reported that 50 to 83% of the plots showed symptoms of esca [6], [9]. For eutypa dieback, the percentages were lower, varying from 30% to 53%. In the same plot, the number of vines showing foliar symptoms of esca was not very high, but this figure increased steadily from 1.04% in 2003 to 3.23% in 2008 (during the same period, eutypa symptoms tended to decrease). However, in order to understand the full complexity of the situation, it is essential to consider five additional points. *(i)* From one year to another the same vines do not necessary express foliar symptoms, so when we consider a period of several years the number of “esca-foliar symptomatic vines at least once” is far more relevant. *(ii)* About 10% of vines of surveyed varieties expressed trunk diseases foliar symptoms, *e.g.* Sauvignon variety in the Aquitaine region. *(iii)* Approximately 13% of French vineyards are unproductive, mainly because of these three diseases. *(iv)* Esca moderately affects the phenolic composition of grapes and decreases the sensory quality of wines, suggesting a particularly dramatic increase in the economic importance of esca if no control methods are found [12]. *(v)* Regarding etiology, the symptoms that occur in the trunk, leaves and berries have been extensively described, indicating that if eutypa dieback symptoms markedly differ from those of esca and of BDA, differentiating between these two latter diseases often proves rather elusive, with Lecomte *et al.* [13] recently providing evidence that foliar symptoms of esca showed transitory phases which overlapped with some BDA descriptions.

Initially, due to the complexity of its symptoms, five different diseases of esca complex were used in the literature: brown wood streaking, Petri disease, young esca, esca and esca proper [14]. However, over the last decade, two major simplifications in disease terminology have been proposed, as reported by Surico *et al.* [14] and Bertsch *et al.* [5]. The first simplification proposed by Surico [15] was that the term “young esca” could be replaced by “grapevine leaf stripe disease” (GLSD), with the term “esca” being reserved for white rot (esca) and esca proper. He went on to propose that, as the same fungi are involved, the term phaeotracheomycotic complex could be used for the three symptomatically different diseases, brown wood streaking, Petri disease and GLSD. Lecomte *et al.* (2012) suggested that a second simplification could be made by not having to separate esca symptoms into mild or apoplectic forms. For the first time, the authors introduced a classification based on a graduated scale of severity, going from leaves showing some discoloration to total vine wilting. As regards the link between foliar and wood necroses, Maher *et al.* [16] recently demonstrated that the foliar expression of esca is associated with several type of internal necroses, which are more extensive than those observed in asymptomatic vines. In symptomatic mature vines, the necroses form a continuum within the plant.

Pathogenic fungi are assumed to be responsible for all these necroses; they grow within the wood, decay it and slowly kill the vines. Development of specific necroses, *i.e.* central necrosis, black punctuated necrosis, white rot and sectorial necrosis, were associated with the multiplication of a few fungi. For instance, *Fomitiporia mediterranea* is predominant in white rot in Europe [17]. Other fungi, such as *Phaeomoniella chlamydospora* and *Phaeoacremonium aleophilum* [18], are often isolated together, but *P. chlamydospora* is the most frequent in central necrosis [1]. Botryosphaeriaceae species are frequent in sectorial necrosis [19], [20]. Certain reports have indicated that these fungi might act simultaneously or successively [8]. For instance, Larignon and Dubos [21] have proposed two processes of wood degradation involving a succession of fungi. The first process, which led to the formation of central, light-colored soft necrosis, included three fungi operating in sequence: *P. aleophilum* and *P. chlamydospora,* followed by *F. mediterranea*. The second process led to the development of light-colored, soft, sectorial necrosis, primarily caused by *Eutypa lata* (also responsible for another vine wood disease, eutypa dieback), followed by *F. mediterranea*. However, the dynamics of necrotic development in woody tissues remains largely speculative [22]. One of the most puzzling points about these fungi is that, although they can cause wood necrosis when they are inoculated on vines, the foliar symptoms are frequently lacking [23]. Koch’s postulate is thus not always validated, and the involvement of other microorganisms in the process of wood degradation is still the subject of speculation. Hofstetter *et al.* [24] have even reported that fungi are not involved in the esca disease, but this is not in line with the literature.

In this context, the objective of our experiment was to produce complementary data that would help in improving our understanding of esca development. So, we compared the fungal microflora inhabiting the apparently healthy wood tissues of young (10 year-old) esca-diseased and healthy vines for three reasons: *(i)* in these plants, the wood tissues of the trunks and rootstocks are mainly non-necrotic and apparently healthy, *(ii)* the mycoflora colonizing the various types of necroses has been extensively described [1], [16], [25] and *(iii)*, as suggested by Maher *et al.* [16], most of these healthy tissues usually became necrotic a few years later, so it would be of great interest to analyze the mycoflora before this event took place. Single Strand Conformation Polymorphism (SSCP) investigations were used to study the structure and dynamics of the fungal communities colonizing symptomatic and asymptomatic vines over a cultural season. The main advantage of this technique is that it can reveal rapid changes in microbial communities, even when their composition is unknown. Additionally, in order to characterize and compare the fungal strains inhabiting the different types of wood tissues of healthy and esca-diseased plants, a classical microbiological approach using plating, completed by DNA-ITS sequencing of the isolated fungi, was used.

## Materials and Methods

### Plant Material and Sampling

Bordeaux Sciences Agro is responsible of Luchey-Halde vineyard. No specific permissions were required for the experimentations we have done. The sampling site was located at the Luchey-Halde vineyard in Pessac-Léognan, (Bordeaux, France). This vineyard has been surveyed for the presence of esca foliar symptoms since its plantation in 2000. Experimentations were carried out on 10-year-old vines of the cultivar Cabernet Sauvignon (*Vitis vinifera*) grafted on rootstock 101 14 MG and planted in a sandy-clay soil. Being interested by the differences between symptomatic and asymptomatic plants, we selected only plants that had previously expressed esca foliar symptoms at least twice over a period of 4 years (2005 to 2009). The control plants never expressed the foliar symptoms.

In order to also monitor the dynamics of the fungal microflora colonizing the wood tissues of vines, 7 asymptomatic and 7 foliar-symptomatic plants were uprooted every 12 weeks over a period of one year. The plants were sampled at 4 dates: in the spring (April 2010), in the summer (June 2010), in the autumn (September 2010) and in the winter (January 2011).

Our sampling method was vine-destructive, as the plants were first uprooted before their various parts were cut longitudinally, *i.e.* cordons, trunks and rootstocks, in order to verify the status of the wood, *i.e.* necrotic or healthy. Except for white rot, which is associated with esca, only non-necrotic tissues were sampled, in order to isolate cultivable fungi and to extract the fungal DNA.

#### Isolation and morphological identification of fungi

All the esca foliar-symptomatic and asymptomatic vines collected in 2010 and 2011, the samples consisted of 15 chips (around 5 mm in length) of non-necrotic woody tissues from the inner and outer parts of trunks and rootstocks.

A surface sterilization method was used in order to eliminate the epiphytic fungi. The wood fragments were immersed in a 5% calcium hypochlorite solution for 30 seconds, washed in sterile distilled water, and dried on a sterile filter paper. For each vine part, 15 sterilized chips were placed on Malt Agar (3 pieces per plate) and incubated at 25°C in the dark. The fungal development was monitored over a 4-week period. When fungal colonies emerged from the wood tissues, mycelial fragments were transferred to new Malt Agar plates. The fungi that did not grow to more than 1 cm around the piece of wood over a period of one month were not isolated.

Whenever possible, the taxonomic identification of the fungi was based on morphological and cultural features, and on examination of fruiting structures and conidia under the microscope. To identify the species of the most abundant genera, and other isolates whose sporulation was not observed, sequencing of the Internal Transcribed Spacer region of the rDNA was performed. It should be noted that the isolates to be sequenced were selected randomly.

#### DNA extraction

For each vine collected, 10 g of non-necrotic tissues were sampled in the inner and outer parts of trunks, in the inner part of rootstocks and cordons, and from the trunk and stock barks. When white rot tissue was identified (only in esca-foliar symptomatic vines), 10 g were also collected. All these samples were ground in liquid nitrogen with a one-ball mill of Dangoumau type, and kept at −80°C prior to DNA extraction.

DNA was extracted from 60-mg aliquots of woody tissues using the Indvisorb Spin Plant mini Kit (Eurobio, France) according to the manufacturer’s instructions. The DNA extracts were then quantified with a nanodrop (ND-1000, Thermoscientific, Labtech), and homogenized at a concentration of 10 ng/µl.

#### SSCP analyses

The pair of primers recognizing the mitochondrial large subunit rDNA gene, ML1-ML2 from White et al. [26], was used for SSCP. DNA was amplified by PCR in an Epgradient Mastercycler (Eppendorf) in a reaction mixture (25 µl final volume) consisting of 1 µl of DNA template (10 ng/µl), 0.2 mM of each dNTP, 1 ng.µl^−1^ of BSA (New Englang BioLabs), 0.2 µM of each primer, Pfu Turbo buffer 1x and 0.05 unit of Pfu Turbo DNA polymerase (Stratagene/Agilent Technologies). The cycling parameters were 95°C for 2 min, followed by 35 cycles at 95°C for 1 min, 56°C for 1 min, 72°C for 1 min and final extension at 72°C for 10 min. The PCR products (∼250 bp) were visualized by 2% TBE agarose gel electrophoresis prior to SSCP analysis.

SSCP analyses were performed on an ABI PRISM 3130 Genetic Analyzer (Applied Biosystems) equipped with four 36-cm long capillaries. One microliter of a PCR product was mixed with 18.8 µl formamide Hi-Di (Applied Biosystems), and 0.2 µl standard internal DNA molecular weight marker Genescan 400 HD ROX (Applied Biosystems). The sample mixture was denatured at 95°C for 5 min and immediately cooled on ice, and then loaded onto the instrument. The non-denaturing polymer consisted of 5.6% POP conformational analysis polymer (Applied Biosystems), 10% glycerol, EDTA buffer 10x (Applied Biosystems) and water. The migration time was set to 2000 seconds, the voltage to 15 kV and the temperature was 32°C. Samples were co-migrated with the fluorescent size standard (GeneScan-400 ROX) to allow comparison of migration profiles between samples. Patterns were then aligned with StatFingerprints (version 2.0) and were gathered in a single numerical database before being statistically described by a global Principal Component Analysis (PCA) with R (version 2.14.2). Each sampling point was calculated by analyzing 250 variables corresponding to the SSCP profile scans of each sample. PCA was performed using the correlation coefficient of Pearson. Variables with a cos2≥0.5 on one of the first third principal components (Dim1, Dim2 or Dim 3) were estimated as sufficiently well represented by the principal plan generated by this PCA.

#### Identification of fungi by sequencing the DNA ITS region

Fungal genomic DNA was isolated from fresh mycelium scraped at the surface of a Malt Agar plate with a sterile tip. Samples were freeze-dried overnight (Alpha 1–4 LO plus, Bioblock Scientific) and then ground with a little glass ball for 1 min at a frequency of 29.9 s^−1^. Four hundred µl of CTAB (1x) were added to each sample. After an incubation at 65°C during one hour, 400 µl of chloroform-isoamyl alcohol (24∶1, v/v) were added and the samples centrifuged for 30 min at 3700 rpm. The aqueous phase was transferred to a new tube, and 200 µl of isopropanol were added. Samples were then kept at −20°C over night for DNA precipitation. After 20 min centrifugation at 3700 rpm, the supernatant was discarded and 500 µl of ethanol 70% were added to wash the DNA. Once the ethanol was discarded, the pellets were air dried and then resuspended in 50 µl of sterile distilled water.

DNA samples were sent to GATC Biotech AG (Konstanz, Germany) for sequencing of the ITS region with the primers ITS1f and ITS4 [26], [27] All sequences were put together and pooled, using CodonCode Aligner software, and assigned to species based on 97% sequence similarity threshold of the ITS region. For species level identification, sequences were subjected to a bulk blastn search against the INSD (International Nucleotide Sequence Databases) as implemented in the PlutoF workbench of the UNITE database [28]. Four hundred and eighty-seven fungal isolates were identified by DNA sequencing of ITS region.

The results of the microbiology study were analyzed using Correspondence Analysis (CA). The data were quantitatively analyzed using the package ade4 of R (version 3.0.1).

Species diversity was then calculated using various indexes: species richness (total number of species observed), the abundance (number of isolates), the Shannon diversity index, H’ [29], [30], the Simpson diversity index, D, evenness, J, obtained by the Shannon equitability index and dominance. Evenness was calculated in order to establish the closeness of equitability of the species present [31]. These indexes were calculated using the package Agricolaea of R (version 2.14.2).

## Results

### Status of the Wood in Esca Foliar-symptomatic and Asymptomatic Vines

**Figure 1:**
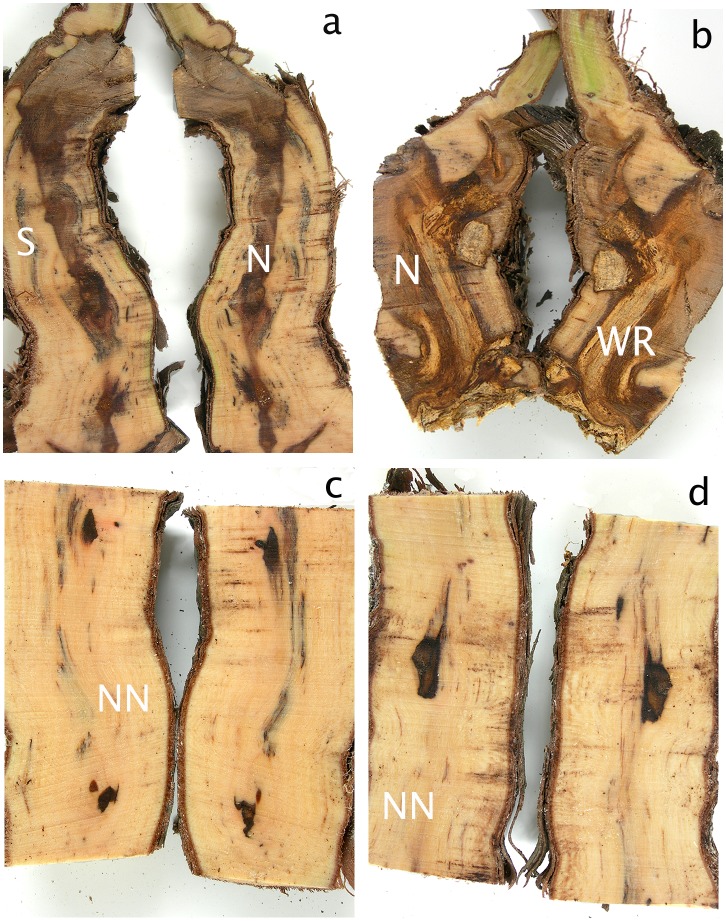
Photographs of longitudinal-sections of different parts of grapevines. (**a**) and (**b**) cordons and (**c**) and (**d**) trunks of plants that had expressed (**b**) and (**d**) or not esca-foliar symptoms (**a**) and (**c**). N = Necrotic tissue, NN = Non-necrotic tissue, S = Stripes, WR = White rot.

Examination of longitudinal sections of vines showed that there were significantly more necroses in the cordons of the esca-symptomatic plants than in the asymptomatic ones (Table 1). Specific necrotic wood tissue, that of white rot, was observed only in vines that had previously expressed esca foliar symptoms, with 79% symptomatic of plants (22/28) having white rot in their cordons (Fig. 1a,b). There was, however, no white rot in the cordons of asymptomatic plants. This difference is highly significant (p<0.0035) in Fisher’s exact test. Non-necrotic tissues were predominant in the trunks (Fig. 1c,d) and rootstocks (not shown) of the esca foliar-symptomatic and asymptomatic plants. Only a small dark necrosis was observed in the centre of the sections, for the two types of plants (Fig. 1c,d).

**Table 1:** Esca-foliar expression of the vines sampled.

Vine plant	Month of sampling	2006	2007	2008	2009	White rot in the cordons
R30 C72	April			X	X	+
R36 C49	April		X		X	+
R37 C70	April		X	X	X	+
R53 C72	April		X	X	X	+
R59 C73	April		X	X	X	+
R60 C37	April		X		X	+
R70 C41	April		X	X	X	+
R37 C51	June		X	X	X	+
R58 C45	June		X		X	
R60 C74	June		X	X	X	+
R67 C54	June			X	X	+
R71 C8	June			X	X	
R8 C8	June			X	X	+
R85 C46	June			X	X	+
R17 C24	September	X	X	X	X	+
R34 C71	September		X		X	+
R36 C61	September		X		X	
R47 C82	September		X	X	X	+
R61 C36	September		X	X		+
R66 C47	September		X	X	X	
R87 C34	September	X	X	X	X	
R25 C50	January		X	X		+
R25 C70	January		X	X	X	+
R28 C75	January		X	X	X	+
R28 C78	January			X	X	+
R3 C52	January	X	X		X	+
R72 C11	January		X	X		+
R8 C51	January	X	X			

Over a 4-year period (2006 to 2009), the year of foliar symptom expression is reported for each plant (noted by x). The presence of white rot in the cordons of vines is indicated by +.

### SSCP Analyses of the Fungal Communities

The mitochondrial 28S rDNA gene was studied to compare the genetic structure and the dynamics of the fungal communities colonizing different types of wood tissue of esca-foliar symptomatic and asymptomatic vines collected over a growing season.

#### Comparison of the fungal communities from the various wood tissues

**Figure 2:**
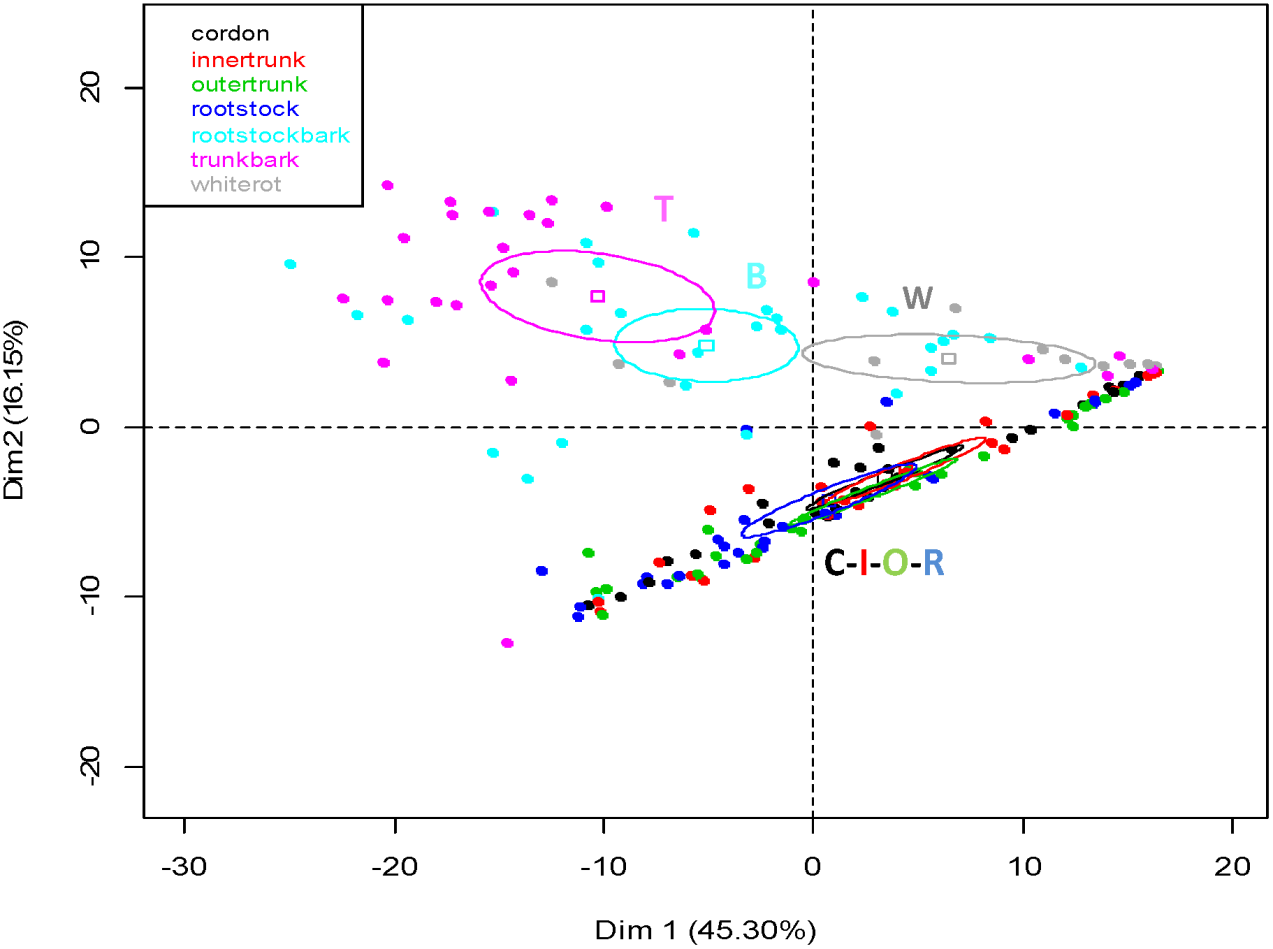
Distribution of the fungal communities on the principal planes defined by the first two axes obtained in the principal component analysis (PCA) of SSCP profiles, using all the wood tissue samples. The variation (%) explained by each axis is given in brackets. Squares correspond to individual SSCPs and ellipses to the 95% confidence intervals calculated for each community. The fungal community of each type of wood tissue is identified by a letter: C = Cordon, I = Inner trunk, O = Outer trunk, R = Rootstock, T = Trunk bark, B = Rootstock bark, W = White rot.

The barks of rootstocks (B) and trunks (T), the non-necrotic wood tissues of rootstocks (R), inner trunks (I), outer trunks (O) and arms (A), and the white rot (W) yielded 208 SSCP profiles, which were compared in Figure 2. For this analysis, symptomatic and asymptomatic vines were pooled for each type of tissue. PCA eingenvalues indicate that the first two principal components, Dim 1 and Dim 2, account for 61.45% of the total fungal variability. Fungal communities differed depending on the kind of tissue they inhabited. However, our results allowed three main types of community structure to be delineated: the first one was associated with barks (B and T), the second with white rot (W), the last one being associated with the non-necrotic wood found in the cordon, trunk and rootstock (C-I-O-R).

#### Comparison of symptomatic and asymptomatic microflora

**Figure 3:**
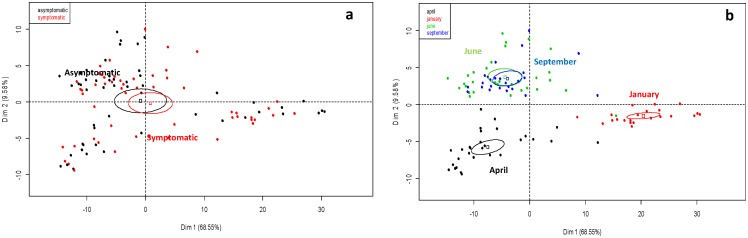
Distribution of the fungal communities on the principal planes defined by the first two axes obtained in the principal component analysis (PCA) of SSCP profiles., using only apparently healthy wood tissue samples. The variation (%) explained by each axis is given in brackets. Ellipses represent the 95% confidence intervals calculated for each community. (**a**) The colours used here represent the profiles obtained from either esca-symptomatic or asymptomatic vines. (**b**) The colours used here represent the profiles obtained at the different sampling dates.

Figure 3a indicates a sample distribution on the principal planes (Dim 1 and Dim 2) generated by the PCA, based on the healthy tissues of the collected vines. Only the individuals representing the fungal communities colonizing the non-necrotic tissues (inner trunks, outer trunks, rootstocks and arms) were considered, and they were pooled for all the sampling dates (April, June, September, January). PCA eingenvalues indicate that the two first principal components, Dim 1 and Dim 2, account for 78.13% of the total data variance. No distinctive patterns were observed for the two types of plants; the fungal community structures were not statistically different, because the ellipses were not clearly separated. Moreover, no influence of the sampling date was observed, as the results were similar when the collection times were considered (Unpublished data).

#### Time sampling comparison

Figure 3b shows the SSCP analyses that were performed on non-necrotic wood tissues collected in symptomatic and asymptomatic vines uprooted at a 12-week interval. A total of 108 SSCP profiles were obtained. Sample distribution on the principal plan generated by the PCA. Evidence was provided from the first PCA axis (Dim1), which accounted for 68.55% of total fungal variability, and also showed that an evolution of the genetic structure of the fungal communities occurred in the wood of vines throughout the year (from April 2010 to January 2011). Examination of the second PCA axis (Dim2), which represented 9.58% of the variance, indicated that those changes could be distinguished according to the season. Indeed, samples collected in spring and autumn were negatively correlated on Dim2, whereas those collected in spring and summer were distributed on the positive y-axis. Moreover, ellipses obtained from the SSCP-profiles of samples collected in June and September displayed significant overlap, thus indicating that the structure of the fungal communities was similar, and that differences were more marked at the beginning (April) and the end (January) of the whole cultivation season.

### Fungal Strain Isolation and Identification

3360 pieces of wood were cultured from the 56 vines sampled, 574 fungal strains were isolated from asymptomatic plants, and 612 fungal strains were isolated from esca-symptomatic plants. Depending on the piece of wood, either no fungus, or upto a maximum of three fungi, were isolated. The highest values of fungi isolated were obtained in April and January, with 205 and 163 isolates, respectively. The lowest values were in June, with around 100 isolates taken from the plants. When the numbers of isolates were compared between symptomatic and asymptomatic vines, differences were observed (p = 0.01049 with Chi-square test) (Unpublished data).

#### Distribution of higher taxonomic units

**Figure 4:**
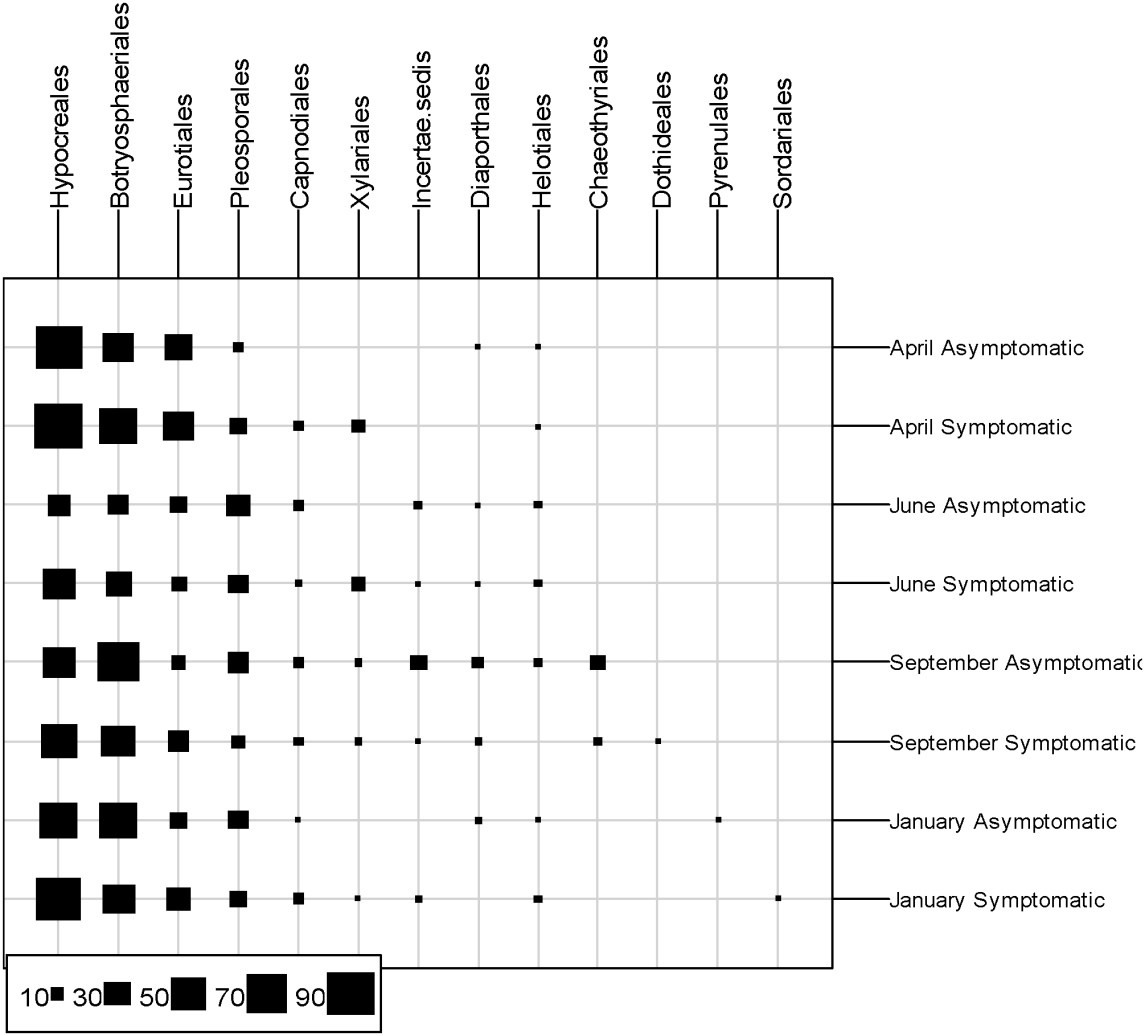
Distribution of Ascomycota orders in apparently healthy wood. Each square represents the number of isolates per kind of foliar symptom and sampling time, pooled over different plant parts.

For each sampling time and for each type of plant, the isolates were ranked in descending taxonomic fashion: division, class, order, family and genus. The Ascomycota were the predominant division, with 92.4% of the total of isolates, followed by the Zygomycota and the Basidiomycota, which were isolated at respectively 7.2% and 0.4%. Figure 4 shows the distribution of Ascomycota orders for each sampling period and type of plant. Thirteen orders were found, of which the most abundant ones were, in decreasing order: Hypocreales, Botryosphaeriales, Eurotiales and Pleosporales. These 4 orders were isolated at every sampling date from the healthy wood of esca-foliar symptomatic and asymptomatic vines. Capnodiales and Heliothales were detected at a lower frequency at eight dates. Dothideales, Pyrenulales and Sordariales were the orders detected only once, September for Dothideales and January for Pyrenulales and Sordariales. The highest number of orders was obtained in September, for the two types of plant, and the lowest in April.

#### Distribution of the fungal genera

**Figure 5:**
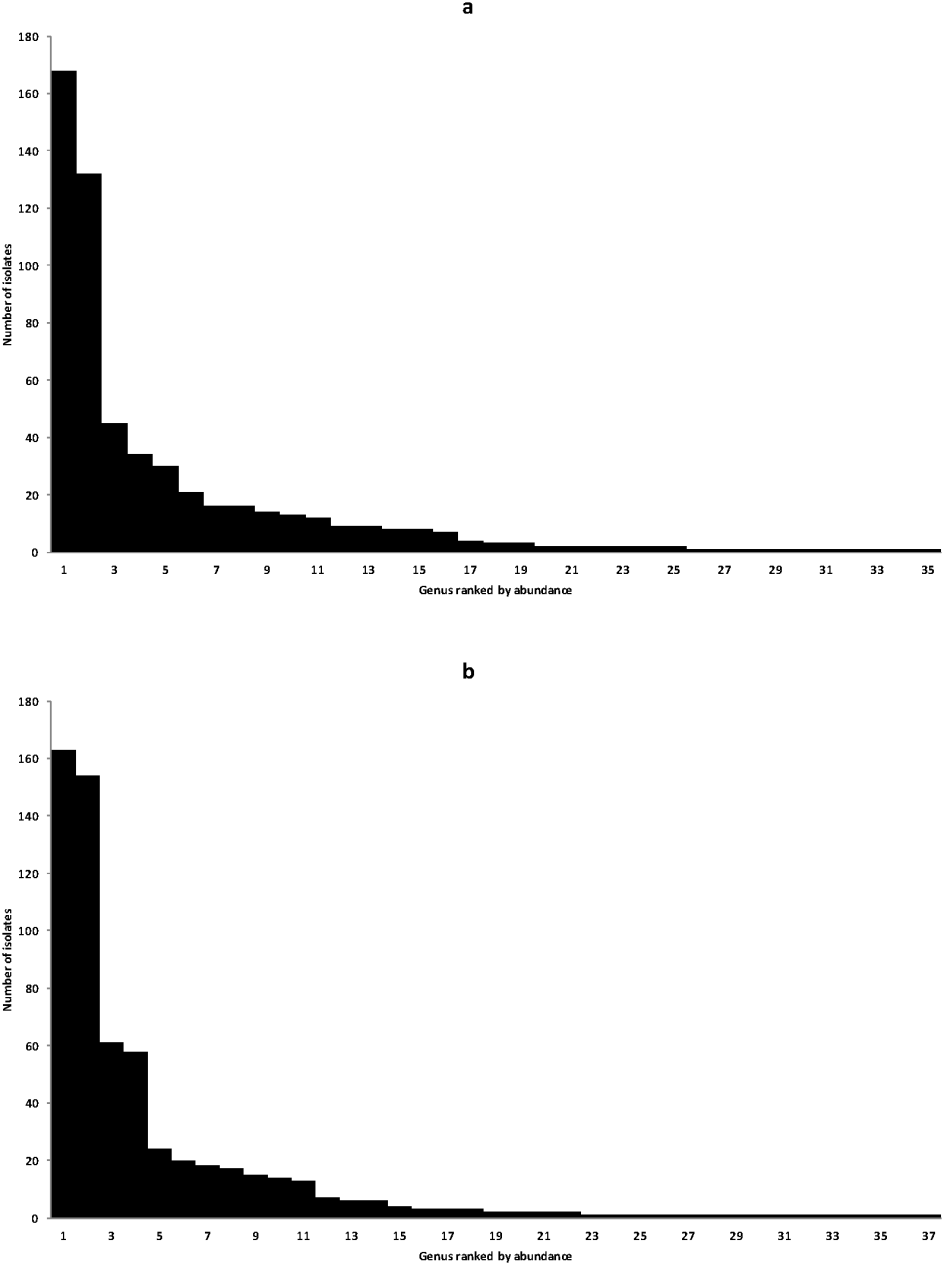
Structure of fungal communities. (**a**) Isolate abundance of the genera isolated from the wood of esca-foliar symptomatic plants. (**b**) Isolate abundance of the genera isolated from the wood of asymptomatic plants.

Figure 5 showed the distribution and frequency of isolates according to the symptomatic plants (Fig. 5a) and the asymptomatic plants (Fig. 5b). Three cases were observed: fungal strains isolated more than twice (plural), twice (double) or only once (single). Globally, 35 genera were isolated from asymptomatic plants, and 37 genera from esca-foliar symptomatic plants. For symptomatic plants, 19 genera were isolated several times, 6 genera twice, and 10 only once. For symptomatic plants, 19 genera were isolated at the different sampling date, 4 genera twice and 14 once. There was no significant difference between the number of plural, double and single genera isolated from symptomatic and asymptomatic plants (P = 0.5035 with Chi-square test).

Table 2 shows the distribution of the fungi according to their class, order, family and genera. A total of 50 genera were identified, the 5 most frequent being *Trichoderma*, *Penicillium* and for the Botryosphaeriaceae*: Botryosphaeria, Diplodia* and *Neofusicoccum*. The fungi isolated from the 50 genera represented 5 classes, 14 orders, 27 families belonging to various lineages of Ascomycota, Basidiomycota and basal fungal lineages.

**Table 2:** Fungal genera isolated from inner and outer wood of rootstocks and trunks of esca-foliar symptomatic and asymptomatic vines over a cultural season.

Class	Order	Family	Fungal genera	April 2010		June 2010		September 2010		January 2011		Totalisolates
				Pl Asy	Pl Sy	Pl Asy	Pl Sy	Pl Asy	Pl Sy	Pl Asy	Pl Sy	
Dothideomycetes	Botryosphaeriales	Botryosphaeriaceae	*Botryosphaeria, Diplodia, Neofusicoccum* To,Ti,Ro,Ri	36	50	16	24	62	42	54	38	322
Sordariomycetes	Hypocreales	Hypocreaceae	*Trichoderma* To,Ti,Ro,Ri	69	61	7	16	23	30	33	56	295
Eurotiomycetes	Eurotiales	Trichocomaceae	*Penicillium* To,Ti,Ro,Ri	26	21	3	5	6	15	10	20	106
	Mucorales	Mucoraceae	*Mucor, Rhizopus* To,Ti,Ro,Ri	23	12	0	4	20	11	8	6	84
Sordariomycetes	Hypocreales	Bionectriaceae	*Bionectria* To,Ti,Ro,Ri	2	17	5	14	3	14	6	13	74
Sordariomycetes	Hypocreales	Nectriaceae	*Fusarium* To,Ti,Ro,Ri	6	5	7	7	11	5	10	3	54
Eurotiomycetes	Eurotiales	Trichocomaceae	*Aspergillus* To,Ti,Ro,Ri	2	16	9	4	2	2	1	2	38
Dothideomycetes	Capnodiales	Davidiellaceae	*Cladosporium* To,Ti,Ro,Ri	0	5	5	2	3	2	1	5	23
Dothideomycetes	Pleosporales	Pleosporaceae	*Alternaria* To,Ti,Ro,Ri	1	2	3	1	8	2	4	1	22
Dothideomycetes	Pleosporales	Montagnulaceae	*Paraconiothyrium* To,Ti,Ro,Ri	0	2	5	7	0	0	4	4	22
Sordariomycetes	Xylariales	Amphisphaeriaceae	*Pestalotiopsis* Ti,To,Ri	0	8	0	7	2	2	0	0	19
Dothideomycetes	Pleosporales	Didymellaceae	*Epicoccum* Ti,To,Ri	0	2	3	2	6	1	3	1	18
Sordariomycetes	Sordariales	Lasiosphaeriaceae	*Arthrinium* To,Ti,Ro,Ri	0	0	3	1	10	1	0	0	15
Dothideomycetes	Pleosporales	Didymellaceae	*Phoma* To,Ti,Ro,Ri	2	2	2	3	1	1	2	1	14
Sordariomycetes	Diaporthales	Melanconidaceae	*Pilidiella* To,Ti,Ro,Ri	1	0	1	1	4	2	2	0	11
Eurotiomycetes	Chaetothyriales	Herpotrichiellaceae	*Phaeomoniella* To,Ti,Ro	0	0	0	0	8	2	0	0	10
Dothideomycetes	Pleosporales		*Pleosporales* To,Ti,Ro,Ri	0	0	2	1	1	3	0	0	7
Dothideomycetes	Pleosporales	Arthropyreniaceae	*Arthopyreniaceae* Ti	0	0	2	0	0	0	0	2	4
Leotiomycetes	Helotiales	?	*Gloeotinia* To,Ro	0	0	2	0	2	0	0	0	4
Ascomycetes	Helotiales	Dermateaceae	*Mollisia* To,Ti,Ro	0	1	0	1	0	0	1	1	4
Dothideomycetes	Botryosphaeriales	?	*Sphaeropsidales* Ti,To,Ro	0	0	0	0	0	0	3	1	4
Ascomycetes	?	?	Ascomycota To,Ri	0	0	1	0	1	1	0	0	3
Agaricomycetes	Polyporales	Polyporaceae	*Trametes* To	0	0	0	0	0	0	0	3	3
	Hypocreales	Hypocreaceae	*Acremonium* To	0	0	0	1	0	1	0	0	2
Leotiomycetes	Helotiales	Sclerotiniaceae	*Botryotinia* To,Ti	1	0	0	0	0	0	0	1	2
Sordariomycetes	Hypocreales	Nectriaceae	*Cylindrocarpon* Ti,Ro	0	0	0	0	0	0	2	0	2
Dothideomycetes	Capnodiales	Davidiellaceae	*Davidiella* Ro,Ri	0	0	0	0	1	1	0	0	2
Dothideomycetes	Pleosporales	Lophiostomataceae	*Lophiostoma* spp. Ro	0	0	2	0	0	0	0	0	2
?	Mortierellales	Mortierellaceae	*Mortierella, Mycena* Ti,Ri	1	0	0	0	0	0	1	0	2
Sordariomycetes	Calosphaeriales	Calosphaeriaceae	*Phaeoacremonium* Ro	0	0	0	0	2	0	0	0	2
Dothideomycetes	Pleosporales	Diothioraceae	*Aureobasidium* To	0	0	0	0	0	1	0	0	1
Leotiomycetes	Helotiales	?	*Cadophora* Ti	0	0	0	1	0	0	0	0	1
Agaricomycetes	Polyporales	Polyporaceae	*Cerrena* Ti	0	0	0	0	0	0	1	0	1
Sordariomycetes	Sordariales	Chaetomiaceae	*Chaetomium* To	0	0	0	0	0	0	0	1	1
Sordariomycetes	Xylariales	Diatrypaceae	*Eutypa* Ri	0	0	0	0	0	0	0	1	1
Leotiomycetes	Helotiales	?	*Hyalodendriella* Ti	0	0	0	1	0	0	0	0	1
Sordariomycetes	Hypocreales		*Hypocreales* To	0	0	0	0	1	0	0	0	1
Dothideomycetes	Pleosporales	Didymellaceae	*Leptosphaerulina* Ti	0	0	0	0	0	0	1	0	1
Dothideomycetes	Pleosporales	Massariaceae	*Massaria* Ti	0	0	0	0	0	0	1	0	1
Dothideomycetes	Botryosphaeriales	Botryosphaeriaceae	*Microdiplodia* Ro	0	0	0	1	0	0	0	0	1
Sordariomycetes	Hypocreales	?	*Myrothecium* Ri	0	0	0	0	0	0	1	0	1
Sordariomycetes	?	?	*Plectosphaerella* Ro	0	0	0	0	0	0	0	1	1
Dothideomycetes	Pleosporales	Sporormiaceae	*Preussia* Ri	0	0	0	0	0	0	0	1	1
Sordariomycetes	Xylariales	Amphisphaeriaceae	*Truncatella* Ri	0	0	0	1	0	0	0	0	1
Dothideomycetes	Pleosporales	Pleosporaceae	*Ulocladium* Ti	0	1	0	0	0	0	0	0	1
Sordariomycetes	Hypocreales	?	*Verticillium* Ro	0	0	0	0	0	0	0	1	1

Pl asy: Asymptomatic plants; Pl sym: Symptomatic plants.

Ti: Inner trunk; To: Outer trunk; Ri: Inner rootstock; Ro: Outer rootstock.

The most frequently isolated genera belong to the class of Sordariomycetes and Dothideomycetes, with a dominance of Sordariomycetes (40% of fungal isolates). Altogether, the two classes represented 77% of all the isolates of symptomatic and asymptomatic plants.

The most abundant isolates collected were: *(i)* Botryosphaeriaceae species (*Botryosphaeria, Diplodia* and *Neofusicoccum* genera) with 168 isolates for asymptomatic, and 154 isolates for esca-foliar symptomatic vines (significant difference with Chi-square test; p = 0.019), *(ii) Trichoderma* spp., with 132 isolates for asymptomatic and 163 for symptomatic (no significant difference with Chi-square test), *(iii) Penicillium* spp., with 45 isolates for asymptomatic plants, and 61 for symptomatic plants (no significant difference with Chi-square test, *(iv) Bionectria* spp. with 16 isolates for asymptomatic plants, and 58 for asymptomatic plants (no significant difference with Chi-square test) and *(v) Fusarium spp.,* with 34 isolates for asymptomatic plants, and 20 for symptomatic plants (no significant differences with Chi-square test). These 7 genera accounted for 68.8% of all the strains isolated from the wood of asymptomatic plants, and for 74.5% from the samples of symptomatic plants.

70% of esca-foliar symptomatic and asymptomatic plants were colonized by Botryosphaeriaceae species and *Trichoderma* spp. For the other species, there were differences between the genera and the number of plants colonized, except for *Penicillium* spp., *Aspergillus* spp. and *Cladosporium* spp., where respectively 50%, 42% and 29% of the both type of plants were inhabited by these fungi. Sixty nine percent of the asymptomatic plants, and 42% of symptomatic ones, were colonized by *Fusarium* spp. It should be noted that *Alternaria* spp., *Arthrinium* spp. and *Epicoccum* spp. colonize more asymptomatic plants than the symptomatic ones.

#### Fungal species isolated from the two types of plants

**Figure 6:**
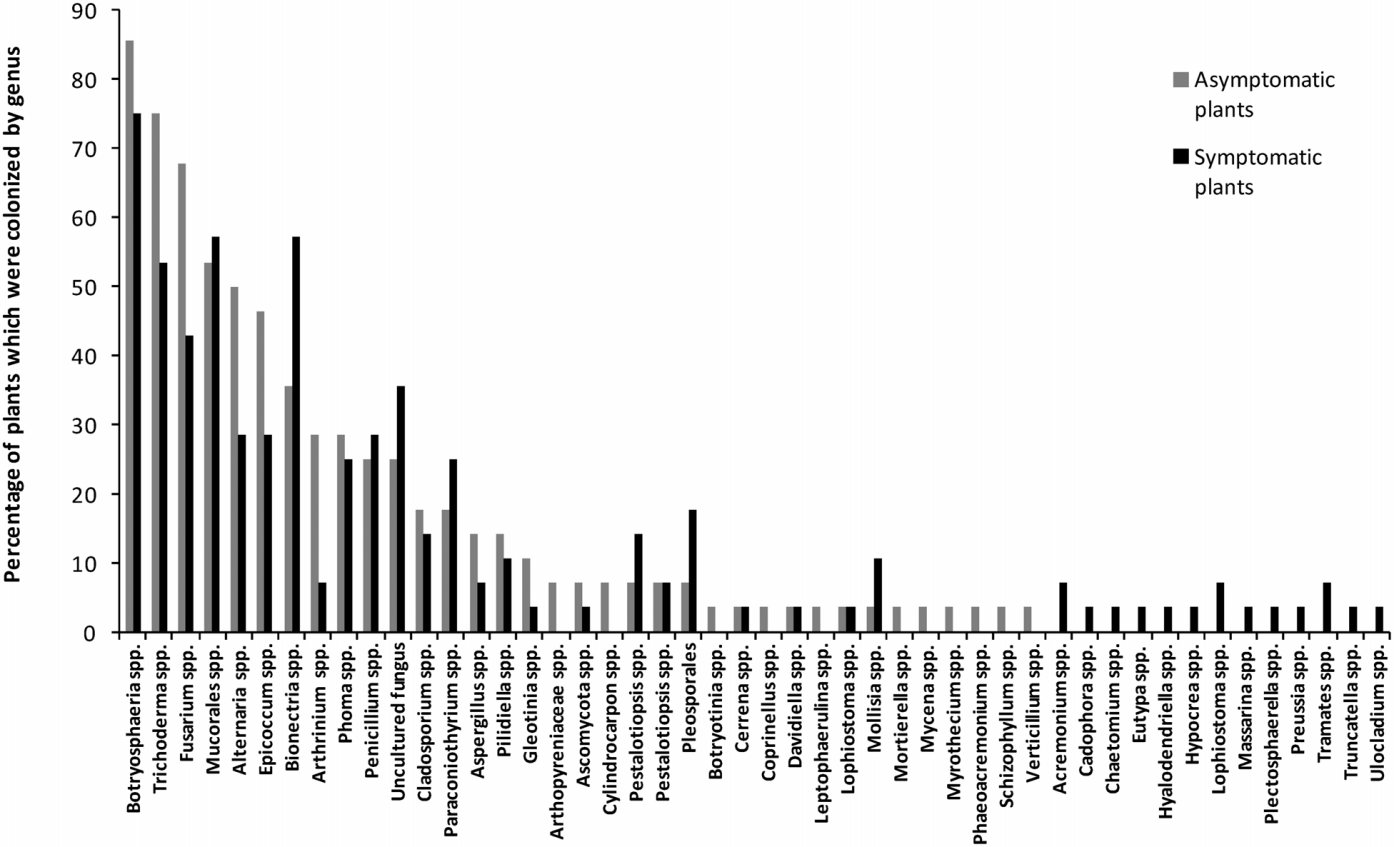
Distribution of the fungal species. The 30 most abundant fungal species isolated from the esca-foliar symptomatic and asymptomatic plants are shown. The percentage represents the number of plants from which the fungi have been isolated.

Figure 6 shows the first 30 most abundant fungal species isolated from the esca-foliar symptomatic and asymptomatic plants. *Diplodia seriata* is the most abundant species. 58% of the symptomatic vines were inhabited by Diplodia seriata, and 75% for the asymptomatic ones. *Fusarium oxysporum* (61%/29% vines inhabited = asymptomatic/symptomatic), *Epicoccum nigrum* (43%/29% = asymptomatic/symptomatic), *T. gamsii* (43%/36% = asymptomatic/symptomatic) and *Arthrinium sacchari* (29%/7% = asymptomatic/symptomatic) were more isolated in asymptomatic plants than in the other type of vine. In contrast, more symptomatic plants were colonized by *Bionectria ochroleuca* (32%/57% = asymptomatic/symptomatic) and *N. parvum* (29%/46% = asymptomatic/symptomatic) than the asymptomatic plants.

### Distribution of the 16 Most Abundant Genera According to the State, Sampling Date and Specific Vine Part

**Figure 7:**
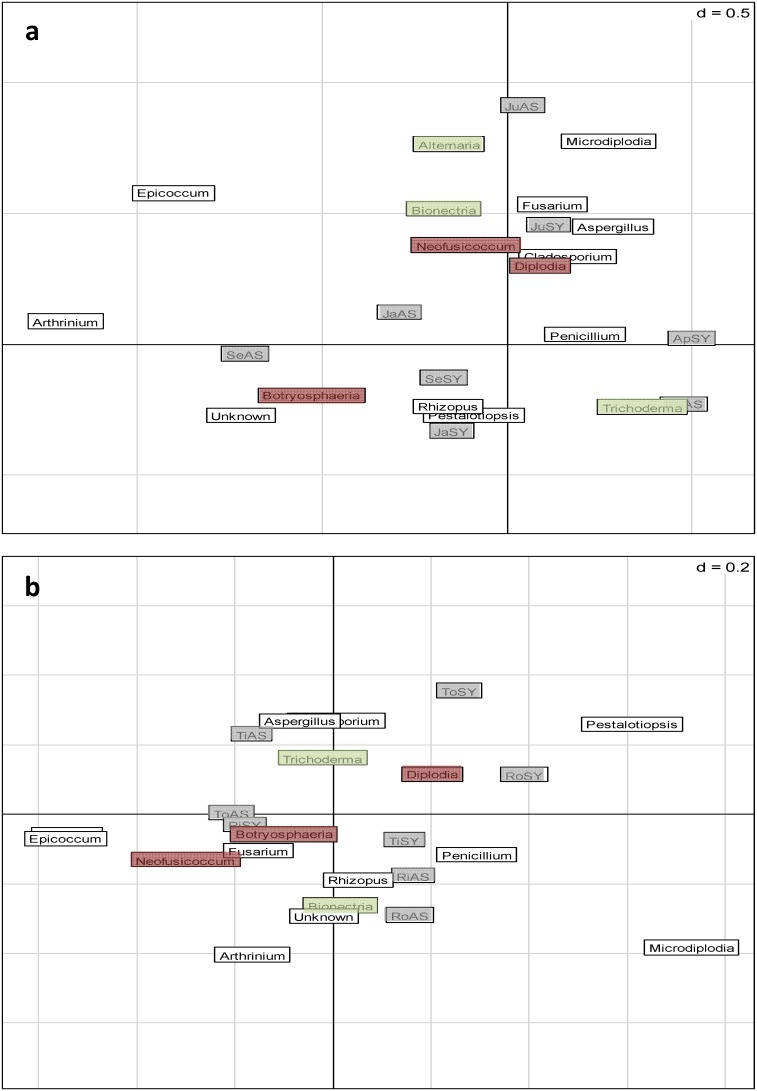
Distribution of the fungal genera isolated from the wood of symptomatic and asymptomatic plants. (**a**) Correspondence analysis of the 16 genera most frequently isolated from woody tissues according to the status of the plants and the sampling date. (**b**) Correspondence analysis of the most 16 genera isolated from woody tissues according to the part of the plants and the sampling date. Av = April; Ju = June; Se = September; Ja = January; SY = Symptomatic; AS = Asymptomatic; Ti = Inner trunk; To = Outer trunk; Ri = Inner rootstock; Ro = Outer rootstock.

Figures 7a and 7b represent 2 Correspondence Analyses which were performed on the 16 most abundant fungal genera isolated, at each sampling time, in the different parts of both symptomatic and asymptomatic plants. Figure 7a shows that, according to the sampling date and type of plant, certain specific genera were associated with particular samples. *Diplodia* sp., *Neofusicoccum* sp., *Bionectria* spp., *Fusarium* spp., *Aspergillus* spp., *Microdiplodia* spp. were isolated more in June from symptomatic and asymptomatic plants. *Rhizopus* spp. and *Pestalotiospsis* spp. were isolated more from symptomatic plants in September and January. Figure 7b represents the link between the 16 most abundant genera, the state and specific particular plant part. *Trichoderma* spp., *Aspergillus* spp. and *Cladosporium* spp. were isolated principally in the inner trunk of asymptomatic plants. *Penicillium* spp., *Rhizopus* spp., *Bionectria* spp., *Arthrinium* spp. and unknown fungi were isolated, in general, from the inner or outer of the rootstock and from the inner trunk of symptomatic plants. The genera *Neofusicoccum*, *Fusarium and Botryosphaeria* were isolated principally from the inner rootstock of symptomatic plants from the outer trunk of asymptomatic plants.

#### Alpha diversity of the fungal genera

Eighty-three species were recorded when all the observations were pooled. To calculate the biodiversity index, the number of species was adjusted for the total number of samples. The fungi used for sequencing were randomly sampled, particularly for plural taxa. Fungal biodiversity from the wood of esca-foliar symptomatic and asymptomatic plants (*H*’) were the same. Depending on sampling time, fungal biodiversity in April (*H*’ sympto = 2.05 and *H*’ asympto = 2.14) was higher than in January (*H*’ sympto = 2.87 and *H*’ asympto = 2.98). Also, dominance was not shown for a relatively large number of species because of the low value of the evenness component (*J*’). The values varied from 0.18 to 0.21 (Table 3).

**Table 3:** Alpha diversity of the fungal genera isolated from the esca-foliar symptomatic and asymptomatic vines.

	Richness (S)	Abundance	Shannon (H)	Simpson (1-D)	Evenness (J)
April symptomatic plants	16	144	2.05	0.49	0.18
April asymptomatic plants	17	114	2.14	0.47	0.19
June symptomatic plants	24	104	2.76	0.36	0.2
June asymptomatic plants	26	128	2.51	0.4	0.2
September symptomatic plants	24	122	2.57	0.39	0.2
September asymptomatic plants	29	163	2.49	0.4	0.2
January symptomatic plants	33	153	2.87	0.35	0.21
January asymptomatic plants	33	141	2.98	0.34	0.21

#### Diversity of haplotypes

After preliminary analyses using Codon Code Software and Mega 5, 487 sequences were selected for phylogenic and statistical analyses. The 487 sequences were assembled in 43 contigs, with one of them grouping the unassembled sequences. Generally, each contig represented one genus or one species. One hundred and four haplotypes were defined from the 43 contigs. When the sequences of one contig were aligned, only a few nucleotides changed for each species. Table 4 illustrates the 6 important contigs in term of sequences: *D. seriata* (72 sequences) followed by *F. oxysporum* (42 sequences), *B. ochroleuca* (33 sequences), *N. parvum* (32 sequences), *T. gamsii* (31 sequences) and *T. atroviride* (19 sequences). These 6 contigs contained 1 to 4 haplotypes. Some haplotypes were detected in the two types of plants for each sampling time; for instance, for H1 and H3 of *D. seriata* and H14 of *T. gamsii*. For the other haplotypes, for which the number of sequences is also generally low, haplotypes were detected only at certain sampling dates.

**Table 4:** Distribution of haplotypes per fungal species for the 6 most abundant colonizing the wood of esca-foliar symptomatic and asymptomatic plants.

Fungispecies	Number ofsequences	Haplotypes	Aprilsymptomaticplants	Aprilasymptomaticplants	Junesymptomaticplants	Juneasymptomaticplants	Septembersymptomaticplants	Septemberasymptomaticplants	Januarysymptomaticplants	Januaryasymptomaticplants
*Diplodia* *seriata*	72	H1 (60)	12	7	6	7	9	5	5	9
		H2 (12)	1	2	1		1	7		
*Fusarium* *oxysporum*	42	H3 (40)	5	3	6	6	4	5	3	8
		H4 (1)						1		
		H5 (1)								1
*Bionectria* *ochroleuca*	33	H6 (24)	4		6	2	2	2	5	3
		H7 (6)	1		1				2	2
		H8 (1)				1				
		H9 (1)								1
		H10 (1)				1				
*Neofusicoccum parvum*	32	H11 (23)		3	7	2	7			4
		H12 (9)	1				3	2	2	1
*Trichoderma* *atroviride*	19	H13 (19)	8	9					1	1
*Trichoderma* *gamsii*	31	H14 (28)	2	1	2	6	5	4	4	4
		H15 (3)	1					2		

In brackets are the numbers of isolates sequenced per haplotype.

## Discussion

The objective of this study was to analyze the fungal communities colonizing the healthy wood tissues that were predominant within relatively young (10 year-old) grapevines. This could help in determining the initial factors that make a healthy wood become necrotic, usually a few years later.

The results, based on the use of SSCP, clearly indicate that the fungal microflora changed over the year. In the rhizosphere of many plants, shifts in the genetic structures of the fungal communities over time were also observed [32]. These shifts were frequently related to the root exudates and physiology of plants, which change over a growing season, shaping the community of microorganisms that metabolize the exudates of the plants. A relatively similar phenomenon within the trunk presumably occurs here. During the period of winter, when the plant is in a latent state, there are no or only low vascular exchanges within the tissues. In spring, the plant produces leaves and grapes, and numerous molecules were transported in the vessels. This source of food probably has an influence on the fungal communities and can, at least partly, explain the shifts observed in the microflora. Additionally, cold temperatures in winter and hot ones in summer may shape the fungal communities.

When the non-necrotic wood of trunks and rootstocks of both esca-foliar symptomatic and asymptomatic plants were studied, their fungal microflora tended to be similar. As expected, the mycoflora were different in the white rot and the non-necrotic tissue for the symptomatic plants. It should be noted that in our study, white rot, a necrosis typical of esca [8], was located only in the cordons of symptomatic plants. Many scientists have already reported that *F. mediterranea* is usually the predominant fungus in this zone [17], [20], [33] and, recently Bruez [34] has also shown, using pyrosequencing analysis that *F. mediterranea* is predominant within the white rot of the grapevines we sampled. So as a specific microflora, presumably dominated by *F. mediterranea*, colonizes white rot, which may explain why that microflora differs from the healthy-wood tissues, as we show by means of SSCP analyses.

The results of the SSCP showed that there were no differences of fungal microflora between the non-necrotic tissues of symptomatic and asymptomatic plants and the results of the cultivable method seemed to show numerous similarities. The number of cultivable fungi we isolated for each sampling date varied for symptomatic and asymptomatic plants from 115 to 280. Ascomycetous fungi represented 92% of the total number of the species obtained. This result is in agreement with reports that have analyzed the microflora colonizing various parts of the vines. For instance, Casieri *et al.* [35] studied the fungi colonizing the wood of disease-free vines, Mostert *et al.* [36], the endophytic fungi associated with shoots and leaves, Tiedemann *et al.* [37] studied those of the vascular system of rootstocks and Gonzalez and Tello [38] those isolated from the leaves, twigs and berries of *Vitis Vinifera*. With the exception of a few samples from Gonzalez and Tello [38], all the endophytic fungi were isolated from healthy vines. Our data were also consistent with other studies dealing with the endophytic fungal communities of woody tissues of different plant hosts [39]–[41]. Although Ascomycota species dominate, some Basidiomycota and Zygomycota were also isolated. In line with Gonzalez and Tello [38], we did not recover any fungus from either the Hymenochaetaceae (*Fomitiporia* spp.) or the Stereaceae (*Stereum* spp.). The main reason is certainly because Basidiomycota, such as *F. mediterranea* or *Stereum hirsutum*, were usually found in the necrotic tissues of grapevine plants. Stone *et al.* [40] mentioned that low proportions of Basidiomycota in endophyte inventories could be due to a bias in the cultural method used to isolate the fungi. Generally, such an approach favours the occurrence of sporulating and fast-growing species rather than wood decay Basidiomycota. In our study, as we analyzed healthy woods, the fact that we isolated numerous Ascomycota would seem reasonable.

At the fungal order level, the high proportion of Hypocreales, Botryosphaeriales, Eurotiales and Pleosporales in the trunk and rootstocks of esca-foliar symptomatic and asymptomatic grapevines suggests they were the main components of the endophytic mycota. Our results differ from those of Hofstetter *et al.* [24] who isolated Pleosporales (32.5%) and Hypocreales (26.8%) from the wood of young vines (sampled from nurseries) and Pleosporales (around 27–28%) and Dothideales (around 15%) from adult plants (15–30 year-old). These differences in fungi microflora may depend on the vine part being sampled and the health status of the wood. Among other explanations: plant age, soil, cultivar, climate, and other environmental factors certainly have an influence on the development of various fungal communities.

For the 12 most abundant genera: *Botryosphaeria, Diplodia, Neofusicoccum, Trichoderma, Penicillium, Bionectria, Fusarium, Mucor, Rhizopus, Aspergillus, Cladosporium, Alternaria,* we observed no significant differences between the healthy wood of esca-symptomatic and asymptomatic vines. Most of these genera were detected in the wood of at least 20% of all the plants. The genera *Botryosphaeria, Diplodia, Neofusicoccum*, have some potentially plant pathogenic species, but the majority of the other genera were non-pathogenic for plants, with some of them being potentially biocontrol agents.

As regards Botryosphaeriaceae species from the genera *Botryosphaeria, Diplodia, Neofusicoccum*, they can cause Bot canker on grapevines [42] but also on many plants like olive trees [43], peach trees [44], [45], almond trees [46], oak [47]. Here, Botryosphaeriaceae species were very frequently isolated from grapevines, being present in 83% and 93% of the esca-foliar asymptomatic and symptomatic plants respectively. The most frequent species was *D. seriata* (57% in symptomatic and 43% in asymptomatic plants). In the literature, contradictory results have been reported: depending on the age of the grapevines and cultivar, Botryosphaeriaceae species could be isolated or not. For instance, Hofstetter *et al.* [24] isolated *D. seriata* from nurseries and adult vines, and *N. parvum* from nursery plants. Krol [48] did not isolate Botryosphaeriaceae species in grapevine canes from nurseries, and Gonzalez and Tello [38] reported that Botryosphaeriaceae species were not abundant in the vines they sampled. Again, diversity in the origin of grapevines resulted in the diversity of Botryosphaeriaceae species, and in the number of strains of each species.

The second, most abundant genus, *Trichoderma*, is well known for its antagonistic abilities to parasite plant pathogenic fungi and to protect many plants, including grapevine [49]–[52]. *Trichoderma* strains were isolated from 75% of esca-foliar asymptomatic and 93% of symptomatic grapevines. The two most frequently isolated species were *T. gamsii* (in 42/35% of asymptomatic/symptomatic plants) and *T. atroviride* (in 25/15% of asymptomatic/symptomatic plants). The other 6 species of *Trichoderma* were isolated to a lesser extent.

In the present study, strains of two haplotypes of *D. seriata*, and of one haplotype of *T. gamsii,* were detected at each sampling time, in both esca-foliar symptomatic and asymptomatic plants. This suggests that these two genera, in addition to being abundant, have specific haplotypes that were able to strongly colonize and persist in the wood of grapevines. Also, the data of the four sampling date show that only two haplotypes of *Diplodia seriata* have been defined, two haplotypes for *Trichoderma gamsii* and one for *Trichoderma* atroviride. There is no difference between the haplotypes of symptomatic and asymptomatic plants. Interestingly, Linaldeddu *et al.* [53] reported that, when they confronted *in vitro* strains of *Trichoderma* and Botryosphaeriaceae species, the mycoparasite was predominant over the development of the pathogen. This finding suggests that such competition occurs in the wood of plants; however, to our knowledge, no investigations have been made to determine if it shapes the two fungal communities at the vineyard.

We found that many other fungi, colonizing at least 20% of plants, may have antagonistic activities. For instance, some strains of *E. nigrum*, *Alternaria alternata*, and *F. oxysporum* were known as useful or promising biocontrol agents. However, it was not possible to postulate that non-pathogenic fungi were always more numerous in asymptomatic grapevines than in esca-foliar symptomatic ones. For instance, the pathogen *D. seratia* was detected in 57% of asymptomatic and 43% of symptomatic grapevines. Furthermore, as the pathogenic status of *F. oxysporum* strains is not known, some may be plant pathogenic. So, when the cultivable mycoflora colonizing the healthy woods of the two types of plants were compared, they could not be statistically differentiated.

In our study, 54% and 51% of total isolates identified from, respectively, asymptomatic and symptomatic plants, represented plural genera. Gonzalez and Tello [38] showed that, in their study, 65% of taxa were isolated more than twice. We isolated many singletons, which can be explained by the competition between the fungi. Some of them are really competitive and grow fast, like *Trichoderma* spp. and Botryosphaeriaceae species. The relative abundance of each endophyte genus reflects an unequal distribution of isolate richness among genera. In fact, in January, the number of different species of isolates was greatest. Also, the low values of evenness underline the fact that few species are abundant in the wood tissue. Unlike the Shannon diversity index (H’) obtained by Gonzalez and Tello [38], *i.e.* 3.17, our indexes were lower. But the diversity we estimated was based on one single grapevine variety (Cabernet-Sauvignon), and on healthy wood samples only.

Nowadays, the scenarios that consist in describing why the healthy wood of grapevines usually becomes necrotic still remains to be written and the whole process demonstrated. In the present experiment, as we observed shifts over a year in the mycoflora colonizing the vine wood, it could be speculated that, over a longer period, fungal community structures could also change. Provided that environmental factors are favourable, pathogenic fungi that have already colonized some parts of the wood of the trunks can multiply and subsequently cause necroses.

In conclusion, this study demonstrates that the mycoflora colonizing the apparently healthy-trunk wood tissues of grapevines changed over the yearly cycle. However, we have not observed that the equilibrium between the potentially plant pathogenic and potentially plant protective fungi colonizing the healthy wood breaks down over time, leading to the development of necroses. Experiments over a longer period, certainly a few years, would certainly be required to observe such changes. We can assume that biotic, *e.g.* bacteria, and abiotic, *e.g.* weather changes, cultural practices and other factors need to be studied in order to understand how those abiotic and biotic factors favor the onset of the pathogenic process.
